# Genetic Testing to Guide Risk-Stratified Screens for Breast Cancer

**DOI:** 10.3390/jpm9010015

**Published:** 2019-03-01

**Authors:** Ava Willoughby, Paul R. Andreassen, Amanda Ewart Toland

**Affiliations:** 1 Departments of Cancer Biology and Genetics and Internal Medicine, The Ohio State University, Columbus, OH 43210, USA; willoughby.89@buckeyemail.osu.edu; 2 Division of Experimental Hematology and Cancer Biology, Cancer and Blood Diseases Institute, Cincinnati Children’s Hospital Medical Center, Cincinnati, OH 45229, USA; 3 Department of Pediatrics, University of Cincinnati College of Medicine, Cincinnati, OH 45229, USA; 4 Comprehensive Cancer Center, The Ohio State University, Columbus, OH 43210, USA

**Keywords:** Risk stratification, risk models, polygenic risk score, hereditary breast and ovarian cancer, breast cancer screening, genetic risk, genetic testing, *BRCA1*, *BRCA2*

## Abstract

Breast cancer screening modalities and guidelines continue to evolve and are increasingly based on risk factors, including genetic risk and a personal or family history of cancer. Here, we review genetic testing of high-penetrance hereditary breast and ovarian cancer genes, including *BRCA1* and *BRCA2*, for the purpose of identifying high-risk individuals who would benefit from earlier screening and more sensitive methods such as magnetic resonance imaging. We also consider risk-based screening in the general population, including whether every woman should be genetically tested for high-risk genes and the potential use of polygenic risk scores. In addition to enabling early detection, the results of genetic screens of breast cancer susceptibility genes can be utilized to guide decision-making about when to elect prophylactic surgeries that reduce cancer risk and the choice of therapeutic options. Variants of uncertain significance, especially missense variants, are being identified during panel testing for hereditary breast and ovarian cancer. A finding of a variant of uncertain significance does not provide a basis for increased cancer surveillance or prophylactic procedures. Given that variant classification is often challenging, we also consider the role of multifactorial statistical analyses by large consortia and functional tests for this purpose.

## 1. Introduction

In the United States, approximately 1 in 8 women will develop breast cancer in their lifetime, and approximately 40,000 women and 460 men die of breast cancer each year [1]. The goal of breast cancer screening is to reduce mortality due to breast cancer. In general, screening is associated with detection of earlier-stage breast cancers and better outcomes [2,3,4,5]. Indeed, in the United States since 1990, the mortality rate of breast cancer has been reduced by 39%, which is due, at least in part, to screening and earlier detection [6]. However, as with any large scale public health interventions, there is a balance between benefits and harms of screening. 

Breast cancer screening includes mammography, a low-dose X-ray of the breast, and for individuals at high-risk, breast magnetic resonance imaging (MRI). There is clear evidence that screening mammography decreases mortality due to breast cancer in women between the ages of 50 and 69 with an estimated risk reduction of mortality of 25–31% [5,7]. Harms related to screening for breast cancer that must weigh into determination of guidelines for screening include false positives and overdiagnoses, which lead to treatment of tumors that otherwise would never have caused problems. Approximately 1 in 9 women undergoing breast cancer screening have a finding on mammogram requiring additional imaging. Of these, over 95% do not have cancer [8]. For women who are tested annually over a period of 10 years, about 50% will have an abnormal finding by mammogram that is not cancer [9,10]. About 7% of these women will undergo a biopsy to follow-up the finding [10]. Abnormalities on screening often lead to additional imaging and breast biopsies; these biopsies are not benign to the woman and can result in distress, scarring, disfigurement, and long-lasting anxiety [11,12,13,14]. False positives on screening mammograms also add to the financial burden of population-based screening.

While DNA damage induced by X-rays could potentially increase the risk of subsequently developing cancer [15], mammograms present a rather low risk for tumorigenesis considering the very low doses delivered each time [16]. Further, we are not aware of any epidemiological studies which indicate that mammograms increase the risk of cancer. Still, since the long-term consequences of low dose irradiation are not well understood, potential benefits of mammogram imaging should be weighed against potential carcinogenic risks [17].

## 2. Screening Modalities and Findings

Mammography works by using low dose X-rays to identify abnormalities in, and changes to, the breast. Mammograms can detect calcifications, which are small build-ups of calcium, and masses that can be tumors. Calcifications are typically classified as microcalcifications which can be associated with tumors and macrocalcifications which are not usually cancer-related [18]. Cysts, or liquid filled sacs, are also detectable by mammogram, but are not associated with cancer. However, cysts are not distinguishable from malignant growths or non-malignant growth such as fibroadenomas; as such, nearly all types of masses require biopsy for definitive diagnosis. More recently, a modification to mammograms, 3-D mammography with digital breast tomosynthesis (DBT) appears to have increased sensitivity for detection of small cancers in breast tissue [19]. This technique generates multiple low dose X-ray images of the breast as a mobile X-ray tube moves in an arc over the breast and a computer merges images to create a three-dimensional representation of the breasts. This technology is thought to both improve sensitivity, particularly for women with dense breasts, and decrease false positives [20]. Breast MRI utilizes a magnet together with radio waves to generate images which are then viewed by computer. It is estimated to have much higher sensitivity for detection of cancer compared to 2-D mammography, but has a wide range specificity of between 20–100% associated with it [21,22]. It is typically not offered to women at average risk of breast cancer, in part due to the high costs, lengthy imaging times, and higher rate of false positives requiring additional follow-up [23]. In contrast, MRI is standard of care in high-risk women and is frequently used for follow-up to evaluate suspicious findings found by mammogram. Advances in MRI imaging of the breast are underway, such as fast abbreviated MRI, which has a decreased image acquisition time relative to standard breast MRI and in small studies shows similar sensitivity with reduced costs [23].

In 1993, a system for reporting mammographic, ultrasound and MRI results, the Breast Imaging Reporting and Data System (BI-RADS), was first developed to increase standardization across providers for identification of suspicious lesions [24]; the most recent version is BI-RADS 5, which was introduced in 2013 [25,26]. Descriptors for findings include those for microcalcification, vascularity, asymmetry, masses, and breast composition. The Breast Cancer Surveillance Consortium (BCSC) model is a more inclusive version of the BI-RADS model for prediction of breast cancer. The BCSC combines mammographic breast density, with factors such as age, race and ethnicity, first-degree relatives with breast cancer, and breast biopsy history. The advantages of BCSC include the ease of identification of factors and ability to be calibrated across different populations [27]. As technology for breast imaging and identifying cancers is improving in both sensitivity and specificity, harms including false-negatives and false-positives will continue to decrease.

## 3. Current Screening Guidelines for Breast Cancer

Current recommended breast cancer screening guidelines vary slightly between agencies [28,29]. The American Cancer Society (ACS) currently recommends that women at average risk begin annual mammograms at age 45 through 54, and can transition to biennial mammograms at age 55 which continue as long as the woman’s health is good and she has a life expectancy of 10 years or greater [30,31]. Women aged 40 to 44 should have the option of starting mammograms. The US Prevention Services Task Force (USPSTF) recommends biennial screening for average risk women beginning at age 50 through 74 years of age [32]. Screening for women aged 40–49 may provide benefit but the risk of false-positive results is higher. The USPSTF notes that the current evidence is insufficient to provide screening guidelines to women over 75.

Screening guidelines change for women with a lifetime risk of breast cancer of 20% or higher. For these high-risk women, an annual breast MRI and mammogram are recommended beginning at age 30 [30,31]. MRI is not recommended for the general population, but is frequently recommended for screening in higher-risk younger women because they have denser breasts for which mammography is not as sensitive [33]. High-lifetime risks are based on family history of breast cancer, carrying a pathogenic variant in a high-penetrance breast cancer susceptibility gene, and other factors such as a prior cancer diagnosis that was treated with radiation therapy to the chest wall [33,34,35]. Genetic testing of highly-penetrant hereditary breast and ovarian cancer genes, discussed below, is used to identify high-risk individuals who would benefit from more aggressive screening. 

## 4. Risk Prediction Models

Breast cancer risk-prediction models may be used to define who will most benefit from heightened screening. In current practice for breast cancer prediction, there is a focus on presenting risk of breast cancer over time and/or likelihood of being a carrier of a *BRCA1* or *BRCA2* pathogenic variant (PV). Many prediction models have been developed to identify individuals at a greater than population risk of developing breast cancer. Models combine many factors such as family history, hormonal and reproductive history, and other personal and environmental contributors to address one or both focuses [36]. 

### 4.1. Models Based on Cumulative Breast Cancer Risk

The Claus, Gail (also known as the Breast Cancer Risk Assessment Tool (BCRAT)), and Rosner-Colditz models all aim to present risk of breast cancer over time [37,38,39]. The Claus model calculates risk using solely the number and degree of relatives affected with breast cancer, as well as their ages at onset. While direct and useful for those with family history, this model is not applicable to women whose risk may be due to lifestyle, environmental, or non-Mendelian genetic risk factors [37]. When only incorporating family history, this may also lead to inaccurate risk prediction in young patients (ages 20–29), because they are less likely to have currently affected close relatives due to their age. In addition, the Claus model only includes those affected with breast cancer, when a family member with ovarian cancer may be just as predictive. Therefore, overall, an individual’s lifetime risk may be underestimated using a family history model.

The Gail and Rosner-Colditz models, on the other hand, while still including family history, focus more on other personal factors. The Gail model includes age at menarche, age at first live birth, and number of previous breast biopsies. For the Rosner-Colditz model, included factors are age at menarche, age at first live birth, birth index (number of children and birth spacing), history of benign breast disease, premenopausal duration, type of menopause, postmenopausal duration, duration of postmenopausal hormone therapy use categorized by type and timing, body mass index (BMI), height, and alcohol consumption. 

The Gail model was one of the first predictive models and is widely used in part due to numerous validation studies within different populations [40]. However, this model only assesses first-degree relatives when incorporating family history, so the portion of risk associated with second-degree relatives is neglected, leading to underestimation of breast cancer risk due to family history [36]. The Rosner-Colditz model’s sizable incorporation of risk factors into breast cancer risk prediction makes this model very unique. On the flip-side, however, the complexity and amount of information needed to be gathered for this model may be a detracting factor to consider when determining the clinical value of this prediction tool.

### 4.2. Models Based on the Likelihood of Being a Carrier of a BRCA1 or BRCA2 Pathogenic Variant

Other models, including BRCAPRO, the Breast and Ovarian Analysis of Disease Incidence and Carrier Estimation Algorithm (BOADICEA), and Tyrer-Cusick (also known as the International Breast Cancer Intervention Study (IBIS)), focus prediction algorithms on genetic risk components [41,42,43,44]. The BRCAPRO and the BOADICEA models aim to present the likelihood of being a *BRCA1* or *BRCA2* mutation carrier. The BRCAPRO model predicts the probability of carrying a *BRCA1* or *BRCA2* mutation, as determined by the status of breast and ovarian cancer, as well as the ages, of the patient’s first and second-degree relatives, and is guided by the prevalence and penetrance of *BRCA1* and *BRCA2* mutation in carriers. Advantages of BRCAPRO include full family history assessment, rather than assessment of just those affected [36]. In addition, the prevalence and penetrance guides may be adjusted for different populations, making this model increasingly universal [41]. However, this model fails to account for pathogenic variants in other breast cancer susceptibility genes and for any potential polygenic component [36].

The BOADICEA model similarly predicts the probability of carrying a *BRCA1* or *BRCA2* mutation, but works by determining the age and status of breast and ovarian cancer in all of the patient’s available relatives, while still being guided by the prevalence and penetrance of *BRCA1* and *BRCA2* mutation in carriers. However, in contrast to the BRCAPRO model, this model also incorporates a polygenic component and calculates the multiplicative effect of many genes that modify breast cancer risk [43]. 

The Tyrer-Cusick model, does not predict the risk of a *BRCA1* or *BRCA2* mutation, but instead aims to present risk of breast cancer over time. It does this by incorporating the risks associated with known *BRCA1/2* pathogenic variants and low penetrance genes, along with personal factors such as age at menarche, age at menopause, nulliparity and age at first childbirth, weight, height and hormone replacement therapy (HRT) [42]. This model became the first to combine genetic factors with hormonal, reproductive, and other personal factors in a comprehensive manner [36]. The Tyrer-Cusick model, while incorporating both *BRCA1* and *BRCA2* mutations, as well as genetic and reproductive factors, has shown to be improved upon with the addition of polygenic risk estimates. In fact, many of these clinical models have shown improvement in predictive values when combining genetic information (Table 1). 

The validation methods for these models include area under the curve (AUC) of the receiver operating curve (ROC), interquartile range odds ratio (IQ-OR), odds ratio *P*_trend_, and net reclassification improvement (NRI). An AUC is historically the most commonly utilized validation method, as it displays the probability that the model is able to accurately distinguish between a case and control. Therefore, a model is labeled as more predictive as the AUC becomes increasingly >0.5. While less commonly utilized, an IQ-OR >1 or odds ratio *P*_trend_ <0.05 shows predictability because it indicates higher association of cases and higher polygenic risk scores. An NRI value indicates the model’s ability to correctly reclassify subjects, which is why is it often used in validating the fitting of a polygenic component to a clinical model.

## 5. Genetic Screening

Breast cancer has been known to run in families since the ancient Greeks [70], and multiple generations of breast cancer within one family was first described in the medical literature in 1866 [70,71]. Over one hundred years later in 1990, the first pathogenic germline variants in the famous tumor-suppressor gene *TP53* were found in affected individuals from families with Li Fraumeni Syndrome [72]. Li Fraumeni syndrome is characterized by a very high risk of early onset breast cancer, sarcoma, leukemia, adrenocortical carcinoma and colorectal cancer [73]. The families in which a *TP53* disease-associated variant was identified had the immediate benefit of being able to identify, through genetic testing for that variant, unaffected individuals in the family who carried the variant and were at an elevated risk of developing cancer. At-risk individuals could then be offered more extensive cancer screening and could consider prophylactic surgeries such as bilateral risk-reducing mastectomy to reduce their risk of cancer-related death. The discovery of *TP53* and other hereditary breast cancer genes, such as *BRCA1* and *BRCA2*, has revolutionized the field of cancer genetics and enabled screening for high-risk women through genetic testing.

### 5.1. Genetic Testing for Hereditary Breast and Ovarian Cancer (HBOC) Genes

The guidelines for who should consider testing to identify PVs in genes that increase risk of breast cancer have changed dramatically since breast cancer genetic testing was first offered clinically in the mid-1990s. Early recommendations for testing were primarily targeted to individuals that had a personal history of early-onset hereditary breast and ovarian cancer (HBOC), and testing was only offered for *BRCA1* and *BRCA2,* two genes associated with an increased risk of HBOC, or *TP53* for individuals that had a family history suggestive of Li-Fraumeni syndrome. Guidelines for genetic testing for *BRCA1*/*BRCA2* differ widely around the world [74]. Current guidelines from the U.S. Prevention Services Task Force (USPSTF) for genetic testing of high-penetrance HBOC genes in unaffected women recommend that women with bilateral breast cancer, a close relative with a diagnosis of breast cancer before 50 years of age, breast cancer in a male relative, family history of breast and ovarian cancer, Ashkenazi Jewish ancestry, or one or more relatives with two *BRCA*-related cancers, meet with a genetic counselor to discuss genetic testing [75]. 

### 5.2. Pathogenic Variants

PVs (also called mutations) are alterations in the DNA that increase the risk of developing disease. For high-risk breast cancer susceptibility genes, PVs are most commonly nonsense or frameshift changes that result in a truncated or degraded protein, but they can also include large deletions, duplications, splice-site alterations or missense variants. Current American College of Medical Genetics (ACMG) and International Agency for Research on Cancer (IARC) guidelines for reporting DNA variants found during clinical testing suggests five classification categories: pathogenic (very strong evidence of disease association), likely pathogenic (variants with strong evidence of pathogenicity), unknown significance (limited and/or conflicting data on pathogenic status), likely benign (variants with strong evidence against pathogenicity), or benign (variants with very strong evidence against pathogenicity) [76,77]. Another term frequently used when describing variants is deleterious; this descriptor is typically used to describe the impact of a genetic variant on protein function, but does not always correlate with disease risk. 

### 5.3. Genes Associated with Hereditary Breast Cancer Risk

Although PVs in several genes have been associated with an increased risk of breast cancer, two genes *BRCA1* and *BRCA2*, appear to account for more cases of hereditary breast (and ovarian) cancer than the others [78,79]. Current estimates are that between 1–7% of unselected breast cancer cases carry a PV in one of these two genes [80,81]. The frequency increases up to 20% of women with a strong family history of early onset breast cancer [82]. Although there is a continuum of risk associated with pathogenic variants in hereditary breast cancer genes, they are typically divided into high-risk (>4-fold increased risk), moderate risk (2–4 fold-increased risk) and low-penetrance (less than 2-fold increased risk) [83]. Certain other genes confer a high-risk of breast cancer (*TP53* and *PALB2*) or a moderate-risk (*ATM*, *CHEK2*, *NF1, PTEN*, *BARD1* and *CDH1*) [84]. A number of other genes have been shown in some studies to confer 2-fold increased risk, some of which are included on clinical breast cancer panels, but the exact lifetime risk and penetrance are not well-established. 

In the past, which genes to test for was determined by what genes were offered, as well as the spectrum and/or pathological characteristics of cancers within the family. These features included PVs in *BRCA1/BRCA2* for breast and ovarian cancer, PVs in *TP53* for sarcoma, breast and other Li-Fraumeni-related cancers, or lobular breast cancer which is more common with *CDH1* PVs. With the advent of large-panel testing being commercially available, many genetics care providers order breast cancer-specific panels or even hereditary cancer panels with over 80 genes for their at-risk patients. The larger breast cancer specific panels generally include the 17 genes listed in Table 2. Most of these encode proteins involved in DNA damage response (DDR) signaling and/or DNA repair via HDR [85,86,87,88]. The other genes encode proteins involved in base excision repair (BER) of DNA, cell cycle regulation and apoptosis in response to DNA damage, cell-cell adhesion, and cytoplasmic signaling [89,90,91,92,93]. A list of PVs for each gene identified through clinical testing can be found at ClinVar [94]. ClinVar [95] is one of several databases that annotates the clinical significance of DNA sequence variants in humans.

The prevalence of PVs in hereditary breast cancer genes in unselected individuals has not been estimated for most of the genes included on clinical testing panels. The best-studied genes are *BRCA1* and *BRCA2* in which the estimated frequency in the general population ranges from 1/200 to 1/500 [82,96,97]. The frequency of a PV is less than 1% of all individuals who have undergone clinical panel testing for hereditary breast cancer [98,99]. However, the frequency of PV-positive individuals is higher in women who already have a diagnosis of breast cancer with about 9–10% of women with breast cancer being found to carry a PV in at least one gene on the panel. Estimates are that about 1.1–1.7% of women with breast cancer have a PV in *CHEK2*, 1.4–2.1% in BRCA1, 1.6–2.2% in *BRCA2*, 0.87–1% in *ATM*, and 0.84–0.87% in *PALB2*, with other genes less than 1% [98,100].

### 5.4. Variants with Different Degrees of Risk/Penetrance

The lifetime risk of breast cancer varies between genes. Current breast cancer risks for carriers of *BRCA1* and *BRCA2* mutations, respectively, are most recently estimated from a prospective cohort to be 72% [95% confidence interval (CI) 65–79%] and 69% (95% CI 61–77%) by age 80 [101]. For *PALB2*, another high-risk gene, the estimated risk of breast cancer by age 70 is 35% (95% CI 26–46%) [102]. PVs in TP53 carry a high lifetime risk of breast cancer of 85% by age 60 years [103]. The large range of cancer risk typically quoted for each gene is because of other factors, including low-penetrance genetic risk alleles and environmental risks that can alter the risk of cancer in PV carriers [104,105]. 

Even within a gene, distinct PVs can confer different risks. In *BRCA1*, some moderately penetrant variants have been identified, including p.Arg1699Gln (c.5096G>A), which has risk of breast cancer to age 70 of about 20% [106,107]. In *ATM*, one missense mutation, p.Val2424Gly (c.7271T>G) confers a higher risk of breast cancer than truncating *ATM* variants (up to 52% in one study; 95% CI 28–80%) and may do so by acting as a dominant negative [108,109,110]. Some of these variants have a functional impact on the protein that they encode but are not as severe as variants that have higher associated lifetime risks.

### 5.5. Screening/Guidelines in High-Risk Individuals

Breast cancer screening guidelines by the National Comprehensive Cancer Network (NCCN) differ according to the gene in which a woman has a PV. For women with a *BRCA* PV, current NCCN recommendations are to receive a clinical breast exam every 6–12 months starting at age 25, an annual breast MRI with contrast beginning at age 25, and an annual breast MRI with contrast and mammogram at ages 30–75. As the lifetime risk of breast cancer is high, many women with PVs in *BRCA1* or *BRCA2* undergo risk-reducing mastectomy, which reduces the risk of breast cancer by 90–95% [111]. Breast screening guidelines for individuals with *TP53* PVs are similar to the *BRCA* genes, except that annual clinical breast exams and MRIs begin at age 20 instead of 25 years. For *PALB2*, *CDH1* and *NF1*, the NCCN recommends an annual mammogram screen and annual MRI beginning at age 30 unless the age of cancer diagnosis within a family is earlier. For moderate risk genes with lifetime risk estimates of 20% or more, the NCCN recommends annual screening mammogram and annual MRI beginning at age 40 unless family history suggests beginning earlier.

### 5.6. Male Breast Cancer

As male breast cancer is quite rare in the general population, there are no breast cancer screening recommendations for men. However, for men who inherit a PV in a high-risk breast cancer gene, the risk of breast cancer is substantially elevated, with lifetime estimates of 4% for *BRCA1* PVs and 7% for *BRCA2* PVs [112]. For male *BRCA* PV carriers, recommendations include monthly breast self-examinations with semi-annual clinical breast exams beginning at age 35 [113].

### 5.7. Cascade Testing

An important aspect of genetic testing is that the results can impact multiple individuals beyond the person being tested. Because pathogenic variants can be inherited, one of the touted benefits of identification of an HBOC PV is that unaffected family members can also be tested for the PV and, if positive, can undergo risk-reducing surgeries and/or more intensive screening/surveillance. However, in practice, cascade testing (testing of relatives for known PVs) has not been as successful as anticipated. One of the difficulties is that the genetic counselor or physician providing results to a patient is not typically the care provider of other family members and, as such, cannot reach out directly to family members. Other barriers to cascade testing include family culture and communication, the impetus for the proband to contact family members and explain the genetic information, and interest/willingness of family members to test [114]. In one successful study of population-identified PV carriers in Israel, almost 94% of carriers (30/32) informed at least one family member. But of those informed, only 48% underwent testing [115]. These results are similar to multiple other studies in Israel showing high rates of communication, but action by relatives to undergo testing ranging from 30–60% in a study setting [115]. Multiple studies are underway looking at new tools to improve the uptake of cascade testing for a variety of genetic diseases. 

### 5.8. Population-based Genetic Screening of High-Risk Breast Cancer Genes

With the advent of next-generation sequencing-based panels and clinical exome and genome analyses, the ability to screen tens to thousands of genes for PVs is feasible. As costs have come down and the benefits of knowing one’s PV status are clear, there have been calls for population-based screening for *BRCA1* and *BRCA2*, as well as other high-risk cancer genes [116]. Knowledge of one’s *BRCA* PV status has been shown to improve health outcomes. For example, all-cause mortality is estimated to be 60% lower in individuals who carry a BRCA PV and follow NCCN recommendations (e.g., prophylactic surgeries) [82,117]. Despite the critical benefits of knowing one’s PV status, key issues related to cost and feasibility of implementation have not been worked out. The main rationale for implementation of genetic screening across the population is that a significant proportion of individuals carrying an HBOC PV do not meet clinical guidelines for testing, which include a strong family and/or personal history of early-onset breast and/or ovarian cancer. A recent study of 1000 individuals with a diagnosis of breast cancer were tested for PVs in genes for HBOC [118]. About 9% had a pathogenic variant in one of the genes on the panel, but only half of those met the testing criteria. Thus, if testing criteria were followed, about 44 women and their family members from this one study would lack critical information related to ovarian cancer risk (in the proband), and breast and ovarian cancer risk in other family members. 

Population-based screening for *BRCA1* and *BRCA2* has been carried out in Ashkenazi Jewish individuals, in the United Kingdom, Israel and Canada, and has been shown to be feasible and affordable [119,120,121]. There seems to be general consensus that population-based testing of *BRCA1* and *BRCA2* in the Ashkenazi Jewish population makes sense given that there are three major PVs found in that population (*BRCA1* 187delAG, *BRCA1* 5285insC and *BRCA2* 6174delT) and they occur in 2-2.5% of unselected individuals [74,122,123,124]. A few non-founder based studies have been published, although others are in process. Findings of a PV variant in *BRCA* were reported from the MyCode Community Health Initiative research study in which unselected individuals in the Geisinger Health System underwent exome analyses [125]. Fifty-five individuals, including 37 who did not know their BRCA status and did not have a BRCA-associated cancer, were found to carry a *BRCA* PV variant. Of these, three individuals were found to carry an early-stage cancer that was treatable. These data suggest that population-based screening has the potential to save lives and may be feasible within a healthcare system. From a financial perspective, screening everyone for HBOC high- and moderate-risk genes via genetic testing, versus using a family and personal history-based criteria for testing, has been estimated to be cost-effective. In one study, quality-adjusted life years and breast cancer prevention, respectively, were estimated at ~$54,770 and 2396 breast cancers per million women screened, when a panel of genes was included [126]. However, not everyone agrees that population screening should be conducted. One of the critiques of population-based testing is how to handle disclosure of variants of uncertain significance (VUS), which are likely to be more frequent than PVs in unselected populations. Also, as genetic counseling is critical for at least disclosure of positive PV results, implementation of population-based testing for HBOC PV is dependent upon a sufficient availability of genetic counselors to provide support to individuals and families found to carry a PV or VUS in a high-risk gene. At this time, there is not a sufficient number of genetic counselors to meet the needs of testing at this level. Other arguments against testing everyone include questions of whether the estimates of cancer risk will be different between families with many diagnoses of cancer, who may have an enrichment in genetic and environmental risk factors, and individuals with no family history [74]. In 2013, the USPSTF officially recommended that only women whose family history suggests that they carry a *BRCA* PV should be tested, and then only after genetic counseling [127]. Studies on how best to implement population-screening (and pay for it) are clearly needed.

One potential strategy that lies between full population-based testing, and testing individuals that have a strong family history or early-age of cancer diagnosis, is to offer HBOC testing to all individuals with a diagnosis of breast or ovarian cancer. One such initiative, Traceback Testing, which is funded by the National Cancer Institute, recommends offering at least *BRCA1* and *BRCA2* testing to all women diagnosed with ovarian cancer identified through pathology or tumor registries, along with follow-up testing and counseling of family members [128]. This approach, however, misses opportunities to prevent cancers, as all of the probands in the study will have had cancer.

### 5.9. Polygenic Risk Scores for Breast Cancer

With the advent of genome-wide association studies (GWAS), sparked by the completion of the Human Genome Project in 2003, came the technological capability and funding to uncover thousands of genetic variants, mainly single nucleotide variants (SNVs), also known as single nucleotide polymorphisms, that correlated with disease predisposition in humans [129]. These variants each typically account for a very low extent of risk, but when many risk variants are considered collectively, their predictive power is much greater, and individuals may accumulate a moderate to high risk of disease similar to that conferred by PVs in HBOC genes. A polygenic risk score (PRS), which combines the effect of many risk variants, is calculated through a weighted sum of the variants’ effects on overall risk of disease in combination with the frequency of that variant in the population [130]. An array of models used to calculate polygenic risk scores for breast cancer have been generated [45,46,49,50,51,52,55,56,57,58,65,67,68,131]. These PRS models show promise for prediction of individuals at lower and higher risk of breast cancer. Studies suggest that the greater the number of SNVs in the model, the greater the predictive ability as measured by AUC calculations of their ROC curves, in which true positive rates are plotted against false positive rates (Table 1). One study of over 94,000 women with breast cancer and 75,000 controls found that women in the highest 1% of a 313 SNV PRS had a lifetime risk of 32.6%, well above that of PVs in genes such as *ATM* and *CHEK2* [45]. Women in the lowest centile of risk had only a 2% estimated lifetime risk of breast cancer. The predictive value of the 313 SNV PRS was better for ER-positive breast tumors as compared to ER-negative breast tumors. This suggests that separate models may need to be run to fully understand a woman’s breast cancer risk. This study has an AUC of 0.630 (95% CI 0.628–0.651) when applied to the subjects as a whole. A major weakness of existing models is the lack of investigation of the more well-studied predictive models in individuals of non-European descent. Recently, there has been an increasing number of polygenic studies on those with non-European backgrounds (Table 1). 

As breast cancer risk is neither genetic alone nor due exclusively to environmental, reproductive or other lifestyle factors, creating predictive models that combine the multitude of non-genetic risk factors along with polygenic risk scores is of critical importance. Several groups have begun to assess this (Table 1). One study combined a PRS based on 92 SNVs with traditional factors and found that addition of the PRS improved the AUC of 0.588 for traditional factors to 0.648 in a combined model [47]. Additionally, future PRS may also be incorporated into risk prediction for individuals that carry PVs in moderate to high-risk breast cancer susceptibility genes. Multiple GWAS have been performed in *BRCA1* and *BRCA2* PV carriers, and PRS scores within this population have been able to better stratify risk [104,132,133]. This concept of modifier genes was studied in *BRCA1* or *BRCA2* PV carriers by Kuchenbaecker et al. (2017) [133]. In a large study, they found that for *BRCA1* PV carriers, the PRS model that predicted ER-negative breast cancers was more predictive than the overall breast cancer model that was more predictive in *BRCA2* PV carriers. Overall, however, they concluded that incorporating PRS and other clinical risk factors, like family history, into a PV carrier’s risk may lead to better care and decision making due to further refinement of breast cancer risk estimates [55]. This was also translated to men with *BRCA1* or *BRCA2* PVs, as Lecarpentier et al. (2017) found that polygenic risk score models created for breast cancer were also indicative of breast cancer risk in men with *BRCA1* or *BRCA2* PVs [134]. They also found that a polygenic risk score model could be formulated for prostate cancer, as well, for men with *BRCA1* or *BRCA2* PVs [134].

Clinical trials have started to evaluate the utility of PRS for breast cancer screening decision-making. In 2016, the WISDOM trial (Women Informed to Screen Depending on Measures of Risk)—a randomized controlled trial with the goal of evaluating annual mammographic screening versus personalized, risk-based screening with respect to the number of late-stage cancers detected, was started. The study has a target goal of 100,000 women, who will be stratified into different screening schedules (i.e., biennial, annual, annual with adjunct MRI, or deferred screening) [135,136]. The risk-based screening arm will incorporate clinical risk factors, breast density, a PRS, and a breast cancer gene panel of moderate-to-high penetrance germline PVs. As different agencies vary in their recommendations as to the optimal time to begin breast cancer screening in women 40–49 years of age, this trial may provide critical information to guide primary prevention for this patient group.

Multiple companies have begun to offer PRS on their breast cancer panels or as a stand-alone test. Myriad Genetics offers a breast cancer risk prediction tool in their commercially available myRisk® Hereditary Cancer test [137], which provides estimates of 5-year and remaining lifetime risk of breast cancer [138]. AmbryScore (Ambry Genetics) offers risk prediction tests to estimate breast risk based on a Tyrer Cusick-like model that includes clinical history (breast: age, ethnicity, family history; prostate: age, ethnicity) plus a PRS that incorporates 100 SNVs [139]. However, the clinical utility of these tests for screening or preventative strategies has not been robustly evaluated. 

## 6. Caveats of Genetic Testing

As discussed above, variants identified in genetic screens that cannot be classified as benign, likely benign, likely pathogenic or pathogenic are designated VUS [76]. Since both *BRCA1* and *BRCA2* have key domains near their C-termini, nearly all nonsense or frameshift mutations that lead to protein truncations are classified as deleterious [140]. Variants present in the last exon of the gene, which are not believed to be subject to nonsense-mediated decay, are a potential exception [141]. In contrast to nonsense and frameshift mutations, missense variants are generally more difficult to classify because they may have more subtle effects on protein function. Many of these missense variants have been observed in only a small number of individuals or families, and thus cannot be classified using standard genetic approaches such as co-segregation of a particular variant with disease in family members.

Novel variants in breast cancer susceptibility genes, many of which are VUS, are frequently identified in genetic screens. As an example, at present, there are approximately 7100 and 9900 distinct germline variants in *BRCA1* and *BRCA2*, respectively, listed in ClinVar, both within and outside exons/introns. Importantly, 37% (~2600) and 46% (~4600) of the *BRCA1* and *BRCA2* germline variants, respectively, listed in ClinVar are VUS. This is most certainly an underestimate of the number of VUS presently identified, since findings may be reported to databases other than ClinVar or may not be reported at all. The fact that >97% and 99% of the VUS listed in ClinVar that affect the coding sequence of *BRCA1* and *BRCA2*, respectively, are missense, underscores the challenges of classifying this category of variants.

It should be noted that findings of VUS limit the power of genetic screens to guide decision making about when to increase breast cancer screening. Although family and personal history are also utilized as part of decisions of when to recommend elevated cancer surveillance, *BRCA1* and *BRCA2* VUS identified in genetic screens do not inform such decisions. The result is that many women who would benefit may not receive the breast cancer screening they need. Thus, risk-stratified breast cancer screening can be enhanced by better classification of variants identified in *BRCA1*, *BRCA2*, and other breast cancer genes.

One promising alternative for classifying VUS are functional tests, based upon expression of a protein which contains the variant in cells with a deficiency for the particular gene. Given the known functions of BRCA1 and BRCA2, such functional tests may be related to homology-directed DNA repair (HDR), cellular resistance to DNA damaging agents, or cellular survival/proliferation in the absence of exogenous DNA damage [140,142]. Since BRCA1 and BRCA2 both have key domains at their C-termini, expression of full-length protein is generally required to functionally test BRCA1/2 variants. Several functional studies of BRCA1 and BRCA2 variants have been conducted by individually expressing variants or individually generating variant alleles utilizing knock-in based strategies [143,144,145,146,147,148,149,150,151]. Notably, this has resulted in the functional characterization of a relatively limited number of variants. Very recently, however, high throughput approaches have been utilized to characterize large numbers of BRCA1 variants in batches [152,153]. In particular, one of these studies utilized saturating mutagenesis, based upon CRISPR-Cas9 mediated gene editing, to functionally characterize nearly all possible amino acid substitutions in each of 13 exons, including variants that have yet to be observed in patients [153]. Such approaches should transform the field by enabling functional characterization of large numbers of variants in other genes, including *BRCA2*. 

At present functional characterization cannot stand alone to determine the pathogenicity of variants in genes such as *BRCA1* and *BRCA2* [77]. The Evidence-Based Network for the Interpretation of Germline Mutant Alleles (ENIGMA) consortium was formed to facilitate variant classification [154]. Members of ENIGMA pool clinical and genetic data to enable classification of some variants. Further, ENIGMA and others have developed multifactorial statistical models that aid in variant classification by considering together clinical and genetic data such as family history, co-segregation of disease in families and histological features of tumors [155,156,157]. The results of functional tests can also be added to multifactorial models to strengthen classification of variants. The hope is, that moving forward, greater numbers of VUS will be classified, thereby enabling more women and their care providers to make informed decisions related to breast cancer risk management.

## 7. Summary and Overview

Here, we have reviewed how genetic testing can be employed to guide risk-stratified breast cancer screens. While earlier detection has reduced the mortality rate for breast cancer [6], there are potential harms by breast cancer screens that utilize mammography or MRI. These include false positives, overdiagnoses, and resulting anxiety in patients. Screening guidelines differ for women with a lifetime risk of breast cancer ≥20%. Since risk prediction models are based on genetic or non-genetic factors, or both, genetic screens are important for achieving optimal risk-stratified breast cancer screening. Genetic screens are increasingly being offered for the identification of pathogenic variants, which increase the risk of developing breast cancer, in *BRCA1*, *BRCA2* and other breast cancer genes. Cascade genetic testing of unaffected family members can identify individuals that may benefit from heightened surveillance for breast cancer, but uptake needs to be improved. While the identification of VUS in genetic screens is prevalent and limits the ability of these screens to guide recommendations for breast cancer screening, extensive efforts are underway to increase the number of variants that are being classified. Notably, in the future, population screens of all women for HBOC high- and moderate risk genes could lead to increased breast cancer surveillance in women that would benefit from this [116,124]. Finally, to enable early detection of cancer, polygenic risk scores are becoming increasingly available clinically as a basis for recommending enhanced breast cancer screening.

## Figures and Tables

**Table 1:** Validations of polygenic and clinical breast cancer risk prediction models.

PRS Model (SNV #)	Validation of PRS	Clinical Factors/ Model	Validation of Clinical Model	Validation of Fitted Clinical Model-PRS	Ancestry	Reference
PRS-313	AUC: 0.63 (95% CI: 0.628 to 0.651)				European	[45]
PRS-100	AUC: 0.61 (95% CI: 0.59 to 0.63)				European	[46]
PRS-92	AUC: 0.623	Multiple factors ^1^	AUC: 0.588	AUC: 0.648	European	[47]
PRS-88	IQ-OR: 1.37 (95% CI: 1.14 to 1.66)	Tyrer-Cusick model	IQ-OR: 1.45 (95% CI: 1.21 to 1.73)	IQ-OR: 1.64 (95% CI: 1.36 to 1.97)	European	[48]
PRS-83	AUC: 0.60 (95% CI: 0.57 to 0.64)	BCSC	AUC: 0.62 (95% CI: 0.59 to 0.66)	AUC: 0.65 (95% CI: 0.61 to 0.68)	Mixed European/ East Asian	[49]
PRS-77	AUC: 0.622 (95% CI: 0.619 to 0.627)				European	[50]
PRS-77	AUC: 0.61 (95% CI: 0.58 to 0.65)	BOADICEA	AUC: 0.66 (95% CI: 0.63 to 0.70)	AUC: 0.70 (95% CI: 0.67 to 0.73)	European	[51]
		BRCAPRO	AUC: 0.65 (95% CI: 0.62 to 0.68)	AUC: 0.69 (95% CI: 0.66 to 0.72)		
		Gail Model	AUC: 0.64 (95% CI: 0.60 to 0.68)	AUC: 0.67 (95% CI: 0.63 to 0.70)		
		Tyrer-Cusick model	AUC: 0.57 (95% CI: 0.53 to 0.60)	AUC: 0.63 (95% CI: 0.59 to 0.66)		
PRS-76	AUC: 0.68 (95% CI: 0.66 to 0.69)	BI-RADS	AUC: 0.66 (95% CI: 0.64 to 0.68)	AUC: 0.69 (95% CI: 0.67 to 0.71)	European	[52]
PRS-76	AUC: 0.64 (95% CI: 0.53 to 0.74)	BCSC	AUC: 0.62 (95% CI: 0.59 to 0.66)	AUC: 0.72 (95% CI 0.62 to 0.82)	East Asian	[49]
PRS-75	AUC: 0.55 (95% CI: 0.53 to 0.58)	Gail Model	AUC: 0.56 (95% CI: 0.53 to 0.59)	AUC: 0.59 (95% CI: 0.56 to 0.61)	African	[53]
		Tyrer-Cusick model	AUC: 0.51 (95% CI: 0.48 to 0.54)	AUC: 0.55 (95% CI: 0.52 to 0.58)		
PRS-75	AUC: 0.581 (95% CI: 0.541 to 0.620)	Gail Model	AUC: 0.652 (95% CI: 0.612 to 0.692)	AUC: 0.658 (95% CI: 0.619 to 0.696)	African	[54]
PRS-71	AUC: 0.59 (95% CI: 0.54 to 0.64)	Gail Model	AUC: 0.55 (95% CI: 0.51 to 0.60)	AUC: 0.61 (95% CI: 0.56 to 0.66)	Hispanic	[53]
		Tyrer-Cusick model	AUC: 0.53 (95% CI: 0.48 to 0.57)	AUC: 0.59 (95% CI: 0.54 to 0.64)		
PRS-67		Gail Model	AUC: Premenopausal: 0.559 (95% CI: 0.541 to 0.577) Postmenopausal not using hormone therapy: 0.555 (95% CI: 0.533 to 0.577) Postmenopausal using HT: 0.580 (95% CI: 0.560 to 0.600)	AUC: Premenopausal: 0.608 (95% CI: 0.590 to 0.626) Postmenopausal not using hormone therapy: 0.611 (95% CI: 0.589 to 0.633) Postmenopausal using HT: 0.619 (95% CI: 0.601 to 0.637)	European	[55]
		Rosner-Colditz Model	AUC: Premenopausal: 0.605 (95% CI: 0.585 to 0.625) Postmenopausal not using hormone therapy: 0.611 (95% CI: 0.586 to 0.636) Postmenopausal using HT: 0.633 (95% CI: 0.609 to 0.657)	AUC: Premenopausal: 0.641 (95% CI: 0.621 to 0.661) Postmenopausal not using hormone therapy: 0.649 (95% CI: 0.624 to 0.674) Postmenopausal using HT: 0.671 (95% CI: 0.647 to 0.695)		
PRS-51	*Odds Ratio P*_trend_: <0.001	Gail Model		NRI: 6.2% reclassification	Singapore Chinese	[56]
PRS-46	AUC: 0.566 (95% CI: 0.517 to 0.614)				Singapore Chinese	[57]
PRS-44	AUC: 0.606				East Asians	[58]
PRS-32	AUC: 0.583 (95% CI: 0.567 to 0.600)	Multiple factors ^2^	AUC: 0.564 (95% CI: 0.547 to 0.581)	AUC: 0.604 (95% CI: 0.588 to 0.621)	European	[59]
PRS-24	AUC: 0.59 (95% CI: 0.55 to 0.63)	BOADICEA		NRI: 23% reclassification	European	[60]
PRS-22	AUC: 0.654 (95% CI: 0.628 to 0.680)				European	[61]
PRS-18	AUC: 0.59 (95% CI: 0.55 to 0.63)				European	[62]
PRS-15	AUC: 0.55 (95% CI 0.52 to 0.59)	Gail Model		≥20% lifetime risk reclassification: +2%	European	[63]
		Claus Model		≥20% lifetime risk reclassification: +3%		
		Tyrer-Cusick model		≥20% lifetime risk reclassification: 0%		
PRS-11	AUC: 0.565 (95% CI: 0.516 to 0.613)				Singapore Chinese	[57]
PRS-10	AUC: 0.597	Gail Model	AUC: 0.580	AUC: 0.618	European	[64]
PRS-9	AUC: 0.557 (95% CI: 0.508 to 0.606)				Singapore Chinese	[57]
PRS-8	*Odds Ratio P_trend_: 2.5 × 10^−15^*	Multiple factors ^3^	AUC: 0.6178	AUC: 0.6295	Chinese	[65]
PRS-7	AUC: 0.587 (95% CI: 0.567 to 0.607)	Gail Model	AUC: 0.557 (95% CI: 0.537 to 0.575)	AUC: 0.594 (95% CI: 0.575 to 0.612)	European	[66]
PRS-7	AUC: 0.5965	Multiple factors ^4^	AUC: 0.6652	AUC: 0.6933	Japanese	[67]
PRS-6	AUC: 0.5979	Multiple factors ^5^	AUC: 0.6338	AUC: 0.6652	Taiwanese	[68]
PRS-5	AUC: 0.574	Age at menarche and first live birth	AUC: 0.638	AUC: 0.658 (95% CI: 0.640 to 0.676)	Chinese	[69]

**Abbreviations**: AUC, Area under the curve; BCSC, Breast Cancer Surveillance Consortium; BI-RADS, Breast Imaging Reporting and Data System; BOADICEA, Breast and Ovarian Analysis of Disease Incidence and Carrier Estimation Algorithm; CI, confidence interval; HT, hormone therapy; IQ-OR, interquartile range odds ratio; NRI, net reclassification improvement; PRS, polygenic risk score; SNV, single nucleotide variant.

**Footnotes**: ^1^ Family history, age at first birth, parity, age at menarche, height, menopausal status, age at menopause, BMI, menopausal hormone therapy use, alcohol consumption, and smoking status. ^2^ Age at menarche, first birth and menopause, count of births, BMI, alcohol consumption, smoking and use of hormone replacement therapy. ^3^ Waist-to-hip ratio, benign breast disease history, age at menarche and first live birth, and family history. ^4^ Age, age at menarche, menopausal status, family history of breast cancer, age at first live birth, BMI, regular exercise, and referral pattern to the hospital. ^5^ Age, BMI, age at menarche, parity, and menopausal status.

**Table 2:** List of genes typically on large breast cancer-specific testing panels.

Gene Name	Name of Encoded Protein	Function
*ATM*	ATM	DDR Signaling
*BARD1*	BARD1	HDR
*BRCA1*	BRCA1	HDR
*BRCA2*	BRCA2	HDR
*BRIP1*	BRIP1/FANCJ	HDR
*CDH1*	E-Cadherin	Cell-Cell Adhesion
*CHEK2*	CHK2	DDR Signaling
*MRE11A*	MRE11A/MRE11	HDR & DDR Signaling
*MUTYH*	MYH	BER
*NBN*	Nibrin/NBS1	HDR & DDR Signaling
*NF1*	Neurofibromin	Cytoplasmic Signaling
*PALB2*	PALB2	HDR
*PTEN*	PTEN	Cytoplasmic Signaling
*RAD50*	RAD50	HDR & DDR Signaling
*RAD51C*	RAD51C	HDR
*RAD51D*	RAD51D	HDR
*TP53*	TP53/p53	Transcription Factor

**Abbreviations**: DDR, DNA damage response; HDR, homology-dependent DNA repair; BER, base excision repair.

## Abbreviations

ACMG, American College of Medical Genetics; ACS, American Cancer Society; AUC, area under the curve; BER, base excision repair; BCRAT, Breast Cancer Risk Assessment Tool; BCSC, Breast Cancer Surveillance Consortium; BI-RADS, Breast Imaging Reporting and Data System; BMI, body mass index; BOADICEA, Breast and Ovarian Analysis of Disease Incidence and Carrier Estimation Algorithm; CI, confidence interval; DBT, digital breast tomosynthesis; DDR, DNA damage response; ENIGMA, Evidence-Based Network for the Interpretation of Germline Mutant Alleles; GWAS, genome-wide association studies; HBOC, hereditary breast and ovarian cancer; HDR, homology-directed DNA repair; HRT, hormone replacement therapy; HT, hormone therapy; IARC, International Agency for Research on Cancer; IBIS, International Breast Cancer Intervention Study; IQ-OR, interquartile range odds ratio; MRI, magnetic resonance imaging; NCCN, National Comprehensive Cancer Network; NRI, net reclassification improvement; PRS, polygenic risk score; PV, pathogenic variant; ROC, receiving operator characteristic; SNV, single nucleotide variant; USPSTF, US Prevention Services Task Force; VUS, variants of uncertain significance; WISDOM, Women Informed to Screen Depending on Measures of Risk.
